# 

**Published:** 1891-04-11

**Authors:** 


### NOTICE TO CORRESPONDENTS.

All MS., Letters, Books for Review, and other matters intended for the Editor should be addressed The Editor, The Lodge, Porchester Square, London, W.

The Editor cannot undertake to return rejected M.S., even when accompanied by stamped directed envelope.

**Notice to Advertisers and Subscribers.**

All Advertisements, Orders for Copies of The Hospital, and Business Communications should be addressed to The Publisher, *not to the Editor*, The Hospital, 140, Strand, W.C.

The Cost of Subscription, post free, is as follows:—Per week, 1½d.; per quarter, 1s. 8d.; per half-year, 3s. 3d.; per annum, 6s. 6d. Foreign:—Per annum, 8s. 8d.; payable, in all cases, in advance.

### General Charities.

[This Column is devoted to an account of work of charitable institutions other than Medical Charities. The Editor will be glad to receive reports or news of work at 140, Strand. *Philanthropy* should be plainly written on the envelope.]

The Annual Court of the Governors of the **CLERGY ORPHAN CORPORATION** for clothing, maintaining, and educating poor orphans of clergymen of the Established Church was held in the last week of February. The Society is in the fortunate position of having an increase of income from subscriptions and donations. They also received a legacy which almost reimbursed them for the sale of stock, which was necessary to meet the expenditure connected with the improvements of the school at Canterbury, where new lass-rooms and a swimming-bath have been erected, and the number of children raised to 207. The age of leaving in the boys' school has now been extended by an extra year.

"Philosophers may sing" of the demoralising influence of giving away food, and say that to give creates a want, but if one is hungry and has no means of satisfying it, what then? Such philosophers have almost invariably the satisfaction of sitting down to an excellent meal three times a day. The **HAM YARD SOUP KITCHEN AND HOSPICE** is appealing for help. This institution is open all the year. The Hospice contains 20 beds for cases of temporary distress, poor convalescents and others of good character who need a helping hand to regain employment. There is no indiscriminate giving away; each family relieved is registered, and is recommended by a Governor; this system prevents such abuses as the sale of tickets and overlapping. In every instance the main object sought is to restore the recipient to a self-supporting position. Boardmen can get a good dinner at the hospice for a penny, and for the same modest sum children can take a dinner home. Funds are urgently needed, and will be gratefully acknowledged by the Secretary, Ham Yard Soup Kitchen, Great Windmill Street, Haymarket, W.

The Managers of the **IRISH DISTRESSED LADIES' FUND** can claim that they have done some substantial good since that Fund was called into existence in 1886 by Mrs. Power Lalor to meet the distress caused through the non-payment of rent in Ireland and other causes consequent upon the unsettled state of that country. The principal objects of the Fund, which have now become well known by name at least, are to provide employment for those ladies who are able to work, and to grant pecuniary assistance to those who, either through age or infirmity, are incapable of helping themselves. Among the latter class are several ladies over 80 years of age, and a few of over 90. It is not so long since that a member of Committee discovered in a cellar in Dublin a lady of 80 years of age whose only means of existence were the small savings of a woman who had once been in her service as lady's-maid, and this case is typical of only too many others as bad or even worse. As so many people have been painfully aware during the past few years, families once in a position of affluence have been cast hopelessly into the direst poverty and destitution, which left for them nothing but the union had not this Fund come to the rescue. We ourselves were acquainted with an aged couple who fifteen years ago were enjoying an income of over £3,000 a-year, who were discovered in the winter literally starving one on each side of an empty grate. The husband was in his eighty-fourth year, and, fortunately, his release came very soon. It was preceded by that of his wife, for whom the strain of adversity had been more than she could bear, and whose mind gave way just before she died. Such cases as these, and they are terribly numerous, must appeal to all people who have hearts and are not despicable cynics. But it is gratifying to know that this Fund has been the means bringing comfort, if not luxury, and some slight rays of renewed hope once more to many whose health and means of support have left them. With the fund has been incorporated "The Association for the Relief of Ladies in Distress through Non-Payment of Rent in Ireland." From its commencement the system adopted has been to give employment, not money, to those able to work, and with this view depôts have been established in London, Dublin, and Cork, from which upwards of six hundred ladies are being supplied with work, and we appeal to Englishwomen who want pretty embroidery or plain needlework done for them to go to 17, North Audley Street and see if they cannot employ their distressed Irish sisters. Other ladies who are willing and able to take situations as companions or governesses are found such employment. Sad to say, subscriptions and donations have been falling off during the past year, from £4,675 in 1889 to £2,906 in 1890, and the Committee plead earnestly to those who have so generously contributed to the Fund in past years to remember that but for their help the recipients of the charity, invalids and sufferers from incurable illness, widows with children, would, through no fault of their own, be in a state of absolute destitution. During 1890 nine of over one hundred pensioners died, and the amount expended in pensions came to £2,058. Ninety-two cases of illness were assisted, and much help was given in meat, &c., to those who needed better food than they could afford. It is, we hope, a happy omen for the future that four of those on the Fund's list have notified that their claims on property having been paid they no longer need assistance. One thing respecting the depôt in North Audley Street is well worthy of praise; it is self-supporting, and has not required any help from the general fund beyond the original grant on its opening. We would remind those generously disposed that underlinen and warm clothing are as acceptable presents to the Committee as money, for the calls during the year on the clothing department are always heavy. Money or clothing will be gratefully acknowledged by the secretary, Lieut.-General Lees, 17, North Audley Street.

### Scraps and Gleanings.

Fossilised vertebrae of an immense whale have been discovered at Whitesands Bay, on Firth of Forth.

The proposed amalgamation of the Cork hospitals, with a view to more economical management, has been abandoned.

In the four years 1886 to 1889 (inclusive), 7,919 cases were treated by M. Pasteur, of whom 79 died, a mortality of only 1 per cent.

Mr. Sewell, a well-known veterinary surgeon in London, has been summoned for cruelty to a number of horses under his treatment.

The eleventh annual report of the Exeter Dental Hospital states that 8,306 patients were treated during 1890, and 4,867 operations performed.

The financial statement of the Stockport Infirmary shows that during 1890 the hospital received a legacy of £3,000 from the late Mr. Mason.

The Worshipful Company of Merchant Taylors have contributed the sum of 200 guineas to the Building Completion Fund of the Royal Free Hospital.

The Chief of the Police at Buenos Ayres has issued an order closing the Salvation Army halls, on the ground that the organisation is not a recognised church.

The number of admissions to the Ilkley Hospital and Convalescent Home in 1890 was 1,122. The receipts of the year were £1,953, and the total expenditure was £1,637.

The Secretary for War has issued an order directing that in future no candidate for admission to the Army Medical Service shall be allowed to compete on more than two occasions.

The dinner in aid of the funds of the North-Eastern Hospital for Children, at which the Duke of Connaught will preside, will be held at the Holborn Restaurant on Friday, May 15th.

The sixth annual report of the Hawick Cottage Hospital is exceedingly satisfactory. The amount of subscriptions is larger than ever before, and the endowment fund has been added to.

Mr. Benjamin L. Cohen has consented to preside at the festival dinner, to be held at the Hotel Metropole on April 15, in aid of the funds of the North London Consumption Hospital.

On March 18 Lord Leigh laid the foundation stone of the new wing of the Warneford Hospital, Leamington. The wing will provide extra accommodation for the nurses and also a new operating room.

The 120th report of the Radcliffe Infirmary states that the subscriptions have decreased by the sum of £200. This deficiency was, however, almost made up by the liberality of the contributions to the Hospital Sunday Fund.

An International Surgical Congress was opened in Paris on March 30th, when over 300 French and foreign surgeons were present. British science was represented by Sir Spencer Wells, Mr. Victor Horsley, Mr. Lawson Tait, and Mr. Godwin.

The report of the Westminster Memorial Cottage Hospital, Shaftesbury, states that the work of the hospital during 1890 was exceptionally heavy and expensive. The receipts amounted to £527 0s. 1d., and the expenditure to £432 9s. 7d., leaving a balance in hand of £94 10s. 6d.

Dr. Thresh, in the paper which he recently read before the Society of Medical Officers of Health, on the relation between Refuse and Diphtheria, attributes to London refuse a peculiar potency for harm, as evidenced by the occurrences of diphtheria held to have been caused by filth.

The heating of the children's ward in the Victoria Hospital, Burnley, has been entrusted to Mr. Birtwistle, who five years ago completed the heating of the other wards in this hospital. The system adopted allows all the steam to return direct to the boiler, no machinery being required for the purpose.

At an amateur performance of "Romeo and Juliet" in Manchester the young man who was playing Mercutio received a fatal wound in the duel with Tybalt. At the inquest the Coroner suggested some degree of negligence in the choice of weapons, but the jury found a verdict of "Accidental death."

The report of the Metropolitan and City Police Orphanage, Strawberry Hill, states that the receipts for the year 1890 amounted to £12,383, of which £4,528 7s. 11d. has been expended on compassionate allowances. Thirty-two boys and 20 girls were admitted during the year to the Orphanage, which now maintains 156 boys and 98 girls.

The comparative statements of pauperism in England and Wales, just issued, in respect of January of this year, show that notwithstanding the severe weather which prevailed during that month, and the increase in the population, the number of paupers in receipt of relief was smaller than the number in the corresponding month in thirty out of thirty-four years comprised in the statement.

The National Society for the Prevention of Cruelty to Children, during the last recorded month of its work, investigated in various parts of the Kingdom 580 complaints of cruelty to children, involving the welfare of 1,331 children. In 77 cases the conduct of the parents or guardians was so hopelessly inhuman that prosecutions had to be instituted, convictions ensuing in 72 of the cases.

The Council of the Society of Arts have offered two gold medals, or two prizes of £20 each, to the Executive Committee of the Congress of Hygiene and Demography for any inventions or discoveries of date subsequent to 1885 exhibited at, or submitted to, the Congress. Under the conditions laid down by the donor, these prizes are to be offered for new methods of obviating or diminishing the risks incidental to industrial occupations.

The will of the late Marchese Vincenzo Bugija, C.M.G., of Valletta, Malta, contains munificent bequests to the institute of public beneficence founded by him under the name of the "Conservatorio Vincenzo Bugija." Five hundred thousand francs is to be set aside for the dispensary branch; 750,000 francs for the reformatory branch; 500,000 francs for founding an asylum for poor old people; and £80,000 for the hospital branch. The testator also bequeathed legacies to other charitable institutions at Malta.

### The Student of Medicine.

[All communications for this Supplement should be addressed to The Editor of The Hospital, at 140, Strand, with the words, "Students' Column," written in the left hand top corner of the envelope.]

### LECTURES: THEIR USE AND ABUSE.

By H. Wolseley Lewis.

*Being a paper read before the Guthrie Society at the Westminster Hospital Medical School on February 12.*

[While this paper treats in part of lectures in general, it is more especially adapted to lectures which form part of the curriculum at a Medical School.—H. W. L.]

Just as it takes more than one person to make an agreement, so also in a lecture it is necessary for the lecturer to have one or more listeners; and since there is usually more than one listener present, so much the more difficult is it for the lecturer to please and benefit all his hearers. I cannot pretend to do more than try to deal with this subject from a hearer's point of view, as I have never had the privilege of being in the position of a lecturer. The word "lecture," as you no doubt know, is derived from the Latin verb "lego," which means "I read aloud," and it is to those lectures more particularly which seem essentially to be based on this original meaning of the word that I want to refer in this paper. It seems to me that lectures nowadays are best used as a means of teaching what cannot readily be learnt elsewhere, and it is with this idea in view that I condemn all those lectures, and they are numerous, which are taken almost verbatim out of those books which stand in the position of text-books to the subject of which the lecture treats. A tale is told of a well-known lecturer on metallurgy, which tells us how, when he had made some particularly interesting and conclusive statement, a facetious student would cry out, "Turn over the page," or "Page number so and so," referring to the chief text-book on the subject; and, indeed, it is only necessary to attend a few of the many lectures, which are delivered on so many subjects in these days, to verify for oneself the fact of how many lectures consist in the almost verbatim recital of the standard works on the subject. If, indeed, these recitals are to stand for lectures, I think that lectures should be arranged on more economical lines; for instance, if a man with a good voice were engaged to read aloud passages out of the best-written text-book, marked by some competent person. In the middle ages, before printing was introduced, much knowledge was handed down by tradition, the members of one generation teaching to those of a succeeding generation by word of mouth what they in their turn had learnt from those of a preceding generation. But now that we have so much and such good printed matter on every subject, there is no need for these gigantic efforts of memory. Of course, it might be said that if the student, whom Horace described as considering—*Non scire, jucundissima vita*—did not have the elements of a subject, as it were, pumped into him in a lecture, he would ignore them altogether, and neither learn them from a text-book, nor perhaps even from a cram-book; but this, I contend, is outside the province of the lecturer, and belongs to the province of the schoolmaster. Lectures were never intended to compel those to learn who "would-not," but rather to offer facilities for learning to those who "would." That this is so is sufficiently conclusively shown by the fact that the lecturer, in so far as he is a lecturer, has no power to compel those to learn who do not wish to, while the schoolmaster, in so far as he is a schoolmaster, is essentially backed up by the much-dreaded cane and other instruments of punishment. I think also that often this reiteration of what the text-books contain may be a reason for finding the student, wearied with much lecturing, placidly sleeping in his place, knowing as he does that he may read and more thoroughly digest the substance of what the lecturer is saying in comfort by the fireside with a pipe. I say more thoroughly digest, for, in reading, the student can vary the pace at will, pausing to think out some more difficult passage, and rapidly reading through the easy ones. Again, it is always possible for him who reads to refer back to some but half-remembered statement, while he who listens must often continue to do so with perplexed and dubious mind. But, you ask, what, then, is a lecturer to lecture on, if he is debarred from repeating what is in the text-books, now that there is a text-book on nearly every subject? My answer is this: Firstly, I think that he should convert himself into a kind of commentary on his subject. There is much in text-books which appears contradictory or even entirely unintelligible to a student who is a beginner and not a master of the subject; again, there are facts, which have to be stated in books, which are necessarily not exhaustive by reason of their size, merely as hard, dry facts, which the lecturer can enlarge upon, look upon from different points of view, and surround with

### The Hospital Advertiser.

THE STUDENT OF MEDICINE—(*continued*).

a halo of interest by apt illustrations from his own and others' experience. I know of nothing which is of more use to the beginner than to hear a lecture in which the salient points are taken up, more widely dilated on, more fully explained, and more intimately discussed by the lecturer, so that that student can go and read his book with more interest and more understanding. Also, there is much which might be brought into lectures in those books, and especially those in foreign languages, which the lecturer in his longer study of the subject may have read, but which it is not possible for the student in his more limited time to consult. Secondly, I would suggest that lectures, accompanied and illustrated by practical demonstration, though they are now given to some extent, have not sufficient stress laid upon them; and this applies especially, I think, to the lectures of medical schools. It will be universally allowed that there can hardly be too much clinical teaching of medicine or surgery, and I use the word in its strictest sense, and not as including lectures held in a room remote from the patient; and not only do medicine and surgery lend themselves particularly to what I may call the "practical" form of lecturing, but also notably "chemistry and physics," physiology, anatomy, histology, and pathology. The reason why I lay such stress upon this "practical" form of lecturing is, that it comes into the useful category of lectures, which teach what cannot readily be learnt elsewhere, which I have already said is the category into which all lectures should come; thus I maintain that no books can supply these practical demonstrations. It is said that "seeing is believing;" I say more, that "seeing is remembering" at any rate, it is certain that it is much easier for most people to remember what they see, than what they only hear. Thirdly, there are a class of lectures which are, I allow, only useful for the most advanced students, or even only a post-graduate course; and these are those in which the lecturer, supposing him to be an authority on his subject, brings forward some of his own views, or some theories which have not as yet been published, except in the transactions of the learned societies, and indeed all those matters which stand at the time on debatable ground, and are not as yet acknowledged facts. Lastly, there are some lectures which are intended to stimulate the interest rather than to convey information, which concern more the emotional and æsthetic than the intellectual sides of our nature; these, however, are not in any way essential to a medical curriculum, but rather apply to art and literature.

(*To be continued.*)

### THE STUDENTS' BOOKSHELF.

### The Manual of Surgical Anatomy.*

At first glance one would imagine, from the size of the volume, that a large amount of important matter must be omitted, but on closer examination there does not appear to be a single point of real surgical interest that is not dwelt upon; and, provided that the student possesses a fair knowledge of general anatomy, no book could be better adapted for examination purposes. The subject of fractures and dislocations is, however, but cursorily dwelt with, "as the aim of the author has been to include only that amount of anatomy which, while requisite for the practice of surgery, is not contained in the works on surgery usually read by students." One can but regret that literature of this type should be necessary, but the present system of examinations absolutely compels the use of "cram" books, and Dr. Hughes has, perhaps, compiled his work in a less objectionable manner than is usual. The work is illustrated with eight full-page plates, and two or three small wood-cuts—they are all essentially diagramatic in character, but show very clearly the points they are intended to elucidate. Especially good are those dealing with the relations of the brachial artery, and of the crural canal; a few more of like nature would have considerably enhanced the value of the book. We might mention by the way that the copy we received contained no fourth plate, unless the two figures on plate three are intended to be regarded as separate plates. There is a useful appendix, consisting of a table of the ossification of epiphyses, and a synopsis of the joints; the latter, however, might with advantage have included their relations, a favourite examination question. The book may be safely recommended for examination purposes to those whose time "is too limited to allow them to study carefully and completely the larger works upon the subject."

* Alfred W. Hughes, M.B., M.S.Edin., F.R.C.S.E., M.R.C.S., Lecturer on Anatomy, School of Medicine, Edinburgh. (E. and S. Livingstone, Edinburgh.)

### The Hospital Advertiser.

### THE STUDENT OF MEDICINE—(*continued*).

### STUDENTS' MEDICAL SOCIETIES.

#### Middlesex Hospital Medical Society.

A meeting was held in the large theatre on Thursday, February 26th. Mr. E. T. Collins, F.R.C.S., gave a demonstration on the histology and pathology of the eye, aided by the limelight lantern. After some beautiful photographs of microscopical specimens of the normal eye had been put upon the screen, Mr. Collins devoted a great deal of attention to the appearances observed in glaucoma, ending up with specimens of the fundus as met with in health and disease. Surgeon Edward Davis, A.M.D., followed by giving an interesting, amusing, and, at the same time, instructive address on the life of an army medical man in India. He urged those who intended entering the services to do so as young as they possibly could, to enter unmarried, and to enter with the intention of looking after one's own particular department of work, and not trying to criticise the work done by the other branches of the army. Mr. Davis spoke very favourably of the feeling which existed between the line officers and the army surgeons, and thought that soon a great deal of the friction would be done away with. He had always found that the other officers treated any surgeon well who treated them well. In conclusion, he compared the Indian Medical Service with the British, pointing out their respective good and bad points. Dr. Coupland proposed a vote of thanks to Mr. Collins and to Surgeon Davis, Mr. J. Bland Sutton seconding it. In putting the vote to the meeting, the President referred to the recent successes obtained by Middlesex men in the examinations for the services, Mr. Deare and Mr. Pridmore passing into the Indian army, Mr. Wade-Brown into the British army, and Mr. E. E. Kershaw into the navy.

### APPOINTMENTS.

At St. George's and St. James's Dispensary, King Street, Regent Street, Edgar A. Hughes, F.R.C.S., has been appointed surgeon. At Guy's Hospital, H. Hodgson, L.R.C.P., M.R.C.S., has been appointed resident obstetric surgeon. At the Queen Charlotte's Lying-in Hospital, Marylebone Road, W. A. Bond, M.A., M.D., B.C. Camb., has been appointed resident medical officer. At the Belgrave Hospital for Children, G. Mansfield, M.B., B.Ch. Edin., has been appointed house surgeon. At the West London Hospital, Hammersmith Road, Malcolm Llewellyn Musgrave, M.R.C.S., L.R.C.P., has been appointed house surgeon, and George Fred. Murrell, M.R.C.S., L.R.C.P., house physician. At the General Lying-in Hospital, York Road, H. Sharland Pope, B.A., M.B., B.C. Camb., has been appointed house physician. At the Teignmouth, Dawlish, and Newton Infirmary, Dispensary, and Convalescent Home, H. F. S. Blucke, L.R.C.P., M.R.C.S., has been appointed house surgeon. At the Infirmary of St. Saviour's Union, Theodore Fisher, M.D. Lond., M.R.C.S., has been appointed assistant medical officer. At Queen's College, Birmingham, W. T. Madin, L.D.S.R.C.S., has been appointed dental tutor. At the West Bromwich District Hospital, Gerald Mansfield, M.B., B.Ch. Edin., has been appointed house surgeon. At the Denbighshire Infirmary, Lewis Owen, M.R.C.S., has been appointed house surgeon. At the Convalescent Hospital at Cheadle, J. E. Platt, M.D. Lond., has been appointed resident medical officer. At the Miller Hospital Patrick Cummin Scott, B.A., M.B. Camb., M.R.C.S. Eng., has been appointed physician. At the Western Dispensary, Edinburgh, Dr. Alex. Thomson has been appointed surgeon. At the Temperance Hospital, Hampstead Road, Helen M. Wilson, M.B. Lond., has been appointed resident medical officer.

### PASS LISTS.

#### Society of Apothecaries of London.

At the March examination there were 158 candidates in arts, of whom three were placed in the first class and 14 in the second class, having passed in all the subjects required for registration as medical student. *First Class.*—C. P. Woodstock, E. M. Leney, and B. F. Henry. *Second Class.*—G. S. Andrewes, C. J. Armson, C. E. Blackstone, F. B. Cardozo, P. Cator, G. A. Crowe, H. E. Fryer, H. Hilliard, J. Ingram, H. E. Izard, W. R. Kingdon, A. E. Malaher, B. Rowlands, and H. J. M. Wyllys. One hundred and eight passed in one or more of the subjects.

In surgery the following candidates passed: E. M. Dobinson, Guy's; W. B. Duck, M.A. Oxon., Oxford University, and St. Mary's Hospital; G. V. M. Gideon, St. Mary's Hospital; C. D. Holmes, Liverpool, University College; S. R. Lane, Glasgow, Middlesex and London Hospitals; W. C. Lattey, St. George's Hospital; W. S. M'Geagh, L.K.Q.C.P.I., St. Thomas's; L. E. Parkhurst, B.A. Oxon., Oxford University and St. Mary's Hospital; A. E. Plumbe, London Hospital; F. Spurr, Sheffield and Middlesex Hospital.

In medicine, forensic medicine, and midwifery the following passed: E. A. R. Covey, St. Bartholomew's; H. H. Crickitt, St. George's Hos-

### The Hospital Advertiser.

### THE STUDENT OF MEDICINE—(*continued*).

pital; G. V. M. Gideon, St. Mary's Hospital; G. Jones, M.A. Oxon., Oxford University and London Hospital; A. J. Lambert, Leeds, Yorkshire College, and Westminster Hospital; A. E. Mayner, M.D., C.M., Montreal University; W. F. H. Newbery, M.D., C.M., Trinity Medical College, Toronto; A. S. Phillips, St. Thomas's; R. D. Waghorn, Westminster Hospital; E. A. Wimberley, Birmingham, Queen's College.

In medicine and midwifery the following passed: T. C. Hughes, Westminster Hospital, and L. Roberts, Cambridge University and St. Mary's Hospital.

In midwifery the following passed: R. W. Brimacombe, Birmingham, Queen's College, and St. Mary's Hospital, and J. S. Newington, Edinburgh University.

To Messrs. Crickitt, Gideon, Jones, Lambert, Mayner, Newbery, Parkhurst, and Wagborn was granted the diploma of the society qualifying for registration and entitling them to practise the three branches of the profession, surgery, medicine, and midwifery.

### University of Aberdeen.

Graduation in Medicine, April 3rd, 1891.

The following candidates receive Degrees in Medicine and Surgery:—

Degree of M.D.—William Alexander, M.A., M.B., C.M., Tarland; Edward Bovill, M.B., C.M., Bengal; Thomas Joseph Compton, M.B., C.M., Norwich; John Craigie, M.B., C.M., Chard; George S. P. Ferdinands, M.B., C.M., Aberdeen; Neil Morrison MacFarlane, M.B., C.M., Preston; Thomas William Adam Napier, M.B., C.M., Egremont, Cheshire; John Moysey Rattray, M.A., M.B., C.M., Frome; John Scott, M.A., M.B., C.M., Manchester; Charles Carter Shepherd, M.B., C.M., Kidderminster; John Taylor, M.B., C.M., Bootle; Thomas Harvey Thomson, M.B., C.M., Campbeltown; Richard Mahoney Townsend, M.B., C.M., Cape of Good Hope. The thesis of George S. P. Ferdinands was considered worthy of highest honours, and that of Neil Morrison MacFarlane of commendation.

Degrees of M.B. and C.M.—Alex. G. Allan, M.A., Portsoy; James Bell, Cults, Aberdeen; Alfred H. Bennett, South Australia; Sidney H. Burnett, Manchester; Wm. H. Clark, Kintore; James D. Crow, M.A., Aberdeen; Patrick W. Diack, M.A., Aberdeen; John P. Farquharson, Aberdeen; Wm. Findlay, M.A., Aberdeen; James W. de Hoedt, Ceylon; Andrew Hunter, Aberdeen; Samuel C. Ironside, Aberdour; John H. Lumsden, Fortrose; George Lyon, Whitehills, Banff; James M'D. M'Kay, Sutherlandshire; George Mackie, Insch; Roderick M. MacLennan, M.A., Ross-shire; John Marnoch, M.A., Aberdeen; Charles Milne, Newhills; Thomas Pimley, Preston; A. Cumming Ross, Craigellachie; David Russell, Banchory; Walter W. Sinclair, Banchory; Charles E. G. Simons, Merthyr Tydfil; George Snell, British Honduras; Wm. H. Stephen, Newhills; Geo. W. H. Tawse, Aberdeen; Clarence E. Wigan, Somerset; John T. Wilson, Glasgow. Robt. L. Brander and William J. Dewar, Arbroath, have passed the examinations for the degrees of M.B. and C.M., but will not graduate until they attain the necessary age.

Graduation Honours.—The following candidates graduate with honours: *Highest Academical Honours*—William Findlay, John Marnoch, Geo. W. H. Tawse. *Honourable Distinction*—Wm. J. Dewar, R. M. MacLennan, John T. Wilson.

The diploma in public health has been conferred on Richard A. S. Eden, M.B., C.M., Aberdeen, William B. Simpson, M.B., C.M., Bedale, Winckworth Tonge-Smith, M.D.

Results of the Professional Examinations for Medical Degrees.

The following Candidates have passed the first division of the first professional examination for the degrees of M.B. and C.M.: Henry J. Baxter, Robert Burgess, Wm. F. Cornwall, Walter P. Crombie, James R. Cruickshank, W. A. G. Farquhar, Wm. S. Fraser, J. L. G. Gillanders, Peter Harper, Lawrence Joseph, James M'Culloch, Donald M'Kenzie, Geo. C. Marke, Robt. D. Moncur, Richard N. Petrie, George A. Pirie, Edward Proudlove, Alex. W. Reid, William Thomson, Robt. M. Trotter, Robt. S. Trotter, Thomas D. Webster.

The following candidates have completed the First Professional Examination: Fred. S. Ainley, Adam Alexander, J. A. Allwood, Frank Beaton, Clifford T. Bell, Robt. H. Boulton, †Alex Brown, Robt. Burgess, Agnus Campbell, K. C. Chetwood-Aiken, A. L. P. Cruickshank, Wm. Cruickshank, A. B. Dalgetty, Alex. Duncan, Wm. C. Edmonston, A. F. A. Fairweather, Donald G. Falconer, G. W. R. Fernando, Hugh Fraser, S. J. C. Fraser, *Frank A. Gill, Duncan Grant, John F. Hall, C. Haslewood, *William Hector, Albert Henderson, Wm. J. Heppard, Peter Howie, John Ingram, Charles Kemp, *John R. Kennedy, Thomas B. Law, Wm. Macdonald, C. G. M'Gregor, Robt. G. M'Gowan, †A. W. Mackintosh, *George Marr, †John Matheson, Charles J. Milne, Robt. Mitchell, †Alfred A. Moore Claud K. Morgan, Chas. L. Morrison, Hector Munro (Fort Augustus), *Hector Munro (Fortrose), P. M. Muttukumaru, Alexander Ogston, *Wilfred W. Pearse, Edward Philip, James F. Philip, William Pieris, A. Ponnambalam, Chas. W. Profeit, Richard A. Slater, Geo. Sturrock, Alex. Thomson, James Thomson, *William Thomson, Geo. E. Watson, †Geo. J. A. Watson, G. A. Williamson, Victor O. Wright.

The following candidates have passed the second professional examination: Norman W. Anderson, William M. Anderson, Arch. C. Barron Geo. G. Bothwell, Thos. Brander, Robt. E. Kerr, Alex. Lamont, R. C. Macdonald, *D. D. Mackintosh, C. W. Marshall, John D. Burnett, Wm. J. Carmichael, James S. Cooper, A. B. Dalgetty, William Dunn, Wm. J. S. Ewan, Alex. Fraser, Thos. H. Galbraith, Alex. Geo. Gall, Geo. Geddes, Geo. K. Gifford, Frank B Graham, Alex. Grant, *John W. Grant, H. T. Hinton, J. W. de Hoedt, Alfred Hogg, Chas. Mitchell, Wm. J. Morton, John G. Pardoe, Arthur W. Paterson, Wm. Paterson, *Wm. R. Pirie, William Ross, *James Rust, Geo. Savege, A. W. Scatliff, George Skeen, *Alfred T. Smith, James Sutherland, Robt. R. Sutter, James Wallace, *John T. West, and Robert Young.

* Indicates that the Candidate has passed "with credit."
† Indicates that the Candidate has passed "with much credit."

